# Benefits and Risks of Moderate Alcohol Consumption on Cardiovascular Disease: Current Findings and Controversies

**DOI:** 10.3390/nu12010108

**Published:** 2019-12-30

**Authors:** Gemma Chiva-Blanch, Lina Badimon

**Affiliations:** 1 Cardiovascular Program ICCC; Institut de Recerca Hospital Santa Creu i Sant Pau—IIB Sant Pau, Sant Antoni Maria Claret, 167, 08025 Barcelona, Spain; 2 CIBER Enfermedades Cardiovasculares (CIBERCV), Instituto de Salud Carlos III (ISCIII), 28029 Madrid, Spain

**Keywords:** alcohol, cardiovascular disease, polyphenols, wine, beer, spirits, myocardial infarction, stroke, hypertension, cholesterol

## Abstract

Alcohol has a hormetic physiological behavior that results in either increased or decreased cardiovascular risk depending on the amount consumed, drinking frequency, pattern of consumption, and the outcomes under study or even the type of alcoholic beverage consumed. However, the vast majority of studies elucidating the role of alcohol in cardiovascular and in the global burden of disease relies on epidemiological studies of associative nature which carry several limitations. This is why the cardiovascular benefits of low–moderate alcohol consumption are being questioned and perhaps might have been overestimated. Thus, the aim of this review was to critically discuss the current knowledge on the relationship between alcohol intake and cardiovascular disease. Besides new evidence associating low and moderate alcohol consumption with decreased risk of cardiovascular disease, several questions remain unanswered related to the concrete amount of safe consumption, the type of alcoholic beverage, and the age-, sex-, and genetic/ethnical-specific differences in alcohol consumption.

## 1. Introduction

Cardiovascular disease (CVD) is the leading cause of mortality in Europe (47% of total mortality) and one of the main causes of death worldwide (31% of all worldwide deaths) [1]. The influence of modifiable risk factors, such as smoking, high blood pressure, dyslipidemia, or poor diet, has been the object of investigation since the 1950s, and studies, such as the Framingham Heart Study [2] or the INTERHEART study [3], have shown that 90% of acute myocardial infarctions (AMIs) are attributable to potentially reversible risk factors, making the reduction of CV risk factors a high priority at the national and global levels. For health systems, alcohol consumption has been a matter of strong debate, because findings from different studies on the effects of alcohol in CVD have been contradictory. Whereas the majority of studies have found that low–moderate alcohol consumption may be beneficial [4]—or at least not harmful [5]—for the CV system by reducing the risk of major adverse CV events (MACE), excessive alcohol consumption increases the risk of CVD [6] and is associated with increased risk of more than 50 diseases [7]. As a matter of fact, alcohol use, besides the associated mental disorders caused by dependence [8], was the seventh leading risk factor for both deaths and disability-adjusted life years in 2016, accounting for 2.2% and 6.8% of total age-standardized deaths among females and males, respectively [9], and it has been identified as a major contributor to the burden of disease all over the globe [7,10].

According to the Dietary Guidelines Advisory Committee (US) [11], if alcohol is consumed, it should be consumed in moderation (≤1 and 2 drinks/day for women and men, respectively) and only by adults of legal drinking age. However, alcohol consumption guidelines vary substantially across the globe: low-risk guidelines range from 10–42 g/day or 98–140 g/week for women and 10–56 g/day or 150–280 g/week for men [12]. In 2016, 32.5% (25% women and 39% men) were current drinkers, and the mean amount of alcohol consumed was 0.73 standard drinks daily for females and 1.7 standard drinks daily for males [9].

Although moderate alcohol intake may have long-term CV benefits, even low consumption may have some risk. Alcohol has a hormetic physiological behavior that results in either increased or decreased CV risk depending on the amount consumed, drinking frequency, pattern of consumption (irregular or heavy/binge drinking, which is not uniformly defined), and the outcome under study [13,14], or even the type of alcoholic beverage consumed [15,16]. Added to this, some factors are critical in the interpretation of the health effects of alcohol consumption in available studies such as the measurement of alcohol consumption (and its misreporting) or the size of the drink (and the respective alcohol concentration). In addition, many individuals do not follow a regular pattern of alcohol drinking, and low–moderate consumption combined with episodes of heavy/binge alcohol drinking may not be beneficial for CVD. Moreover, alcohol intake in the majority of epidemiological studies is measured only once and through food frequency questionnaires or from quantity–frequency measures which may underestimate alcohol consumption, because the validity of self-reported alcohol intake has been questioned due to the fact of fear of stigmatization [10]. These questions need to be further addressed in epidemiological trials and alcohol exposure needs international standardization, because the cut-off points for the alcohol intake categories differ substantially among studies.

Considering all these limitations, the CV benefits of low–moderate alcohol consumption are being questioned, and it is considered that they might have been overestimated. Thus, the aim of this review was to critically discuss current knowledge on the relationship between alcohol intake and CVD. 

## 2. Moderate versus Heavy Alcohol Intake

To analyze the effects of alcohol consumption in CVD and/or overall health, alcohol consumption levels must be defined. As summarized in Table 1, the US National Institute on Alcohol Abuse and Alcoholism defines drinking levels as low-risk drinking, moderate alcohol consumption, binge drinking, and heavy alcohol use [17], although some studies have their own classification. Moderate consumption of alcohol is referred to as regular daily consumption, whereas low intake, at the same maximum amount of alcohol intake as moderate consumption, is referred to as occasional and alternated with some days with no alcohol consumption. In this review, this classification will be used, and low-risk consumption will be referred to as low consumption.

As it will be further discussed, the relationship between CVD and alcohol intake is complex and responds to a hormetic behavior as reflected by U- or J-shaped relationships, with low–moderate intake being more protective than abstention or abusive drinking. However, and besides the drinking category, it is extremely important to consider that subjects in the non-drinker’s category (i.e, teetotalers) generally comprise both never drinkers and ex-drinkers (i.e., sick-quitters). Former drinkers may have ceased alcohol consumption because of serious or chronic illness, previous alcohol abuse, prodromal symptoms before clinical manifestations of major events, and/or because of prescription medications incompatible with alcohol consumption. It has been reported that teetotalers have significantly more prevalent CV risk factors than low to moderate drinkers which would overestimate the health benefits of alcohol intake [18,19,20] and bias or underestimate recent alcohol intake which might explain, in turn, the cross-study heterogeneity found in meta-analyses.

In addition, the main body of studies on alcohol’s impact on CVD relies on cohorts above 35 years of age which may bias the effect of lifetime drinking. It has been shown that drinking patterns vary during lifetime, whereas heavy intake of alcohol peaks in one’s 20s and is accompanied with episodes of binge drinking (in which, by coincidence, CVD controls are rare [21]) and decreases to minimum in one’s 40s. In fact, heavy and irregular binge drinking (>60 g alcohol) is associated to increased arterial stiffness [22], even at early ages [23], and with a 45% increased risk of ischemic heart disease compared to moderate drinking [24]. Therefore, subjects with heavy/binge regular drinking in their 20s are more prone to be abstainers above 35 years of age; therefore, the CV risk among these subjects—considered teetotalers in several studies—may be high compared to lifetime moderate drinkers because of underestimated heavy/binge drinking patterns during adolescence and early adulthood [21,25] and not because of the potential protective effects of moderate alcohol consumption. 

A cross-sectional study showed that low, moderate, and heavy drinkers had significantly lower allostatic load scores, which includes measures of cardiovascular, autonomic, metabolic, neuro-endocrine, and immune systems [26], than lifelong abstainers or former low drinkers [27], and that score is associated with lower risk of CVD and all-cause mortality [28]. However, it is undeniable that chronic heavy and binge drinking is harmful at all levels. In the CV system, it has been extensively associated with increased risk of diabetes, hypertension, ischemic and hemorrhagic stroke, cardiomyopathy, atrial fibrillation, and with increased mortality after an (-but not with increased risk of) AMI [29] probably because of the hypertension provoked by alcohol [14,30]. In addition, irregular heavy drinking (to similar monthly amounts of alcohol consumption as moderate drinkers) is also associated to increased risk of overall CVD because of its negative impact on the lipid profile, arrhythmias, and increased blood pressure [10]. Nevertheless, even in countries with the highest intake of alcohol per capita, chronic heavy and irregular binge drinkers account for the minority of drinkers [31]. 

Besides the amount of alcohol ingested, the accompaniment may be of importance. When consumed with food or within meals, alcohol intake is associated with lower risk of acute AMI [13]. However, it has been recently reported that when consumed with energy drinks, they can induce an elevated risk of CVD, because energy drinks may influence consumption of higher amounts of alcohol, decrease the perception of intoxication, and induce impaired driving, risky sexual behavior, and alcohol dependence [32], especially in teenagers who are also more prone to binge drinking [33].

## 3. Acute versus Chronic Alcohol Consumption

The CV (and overall) health effects of drinking are both acute and chronic (accumulative) and are strongly determined by the quantity and pattern of alcohol intake. In turn, the acute response to alcohol may also be determined by drinking habits and alcohol tolerance [14].

Acute and excessive/binge alcohol intake is associated with the development of acute arrhythmias and ventricular function depression [34], although the clinical manifestations of alcohol-induced heart damage are more frequently presented after more than 10 years of alcohol abuse [35]. In fact, the patterns of drinking, more than the total amount consumed, determine CVD risk and, more specifically, the risk of AMI [36]. Along this line, it has been observed that irregular binge drinking (1–2 days per week with a similar total amount of alcohol as moderate drinking) is associated with increased CV risk compared to subjects drinking the same weekly amount on a regular daily basis (moderate consumption) [13].

In a recent meta-analysis, including 23 studies related to alcohol consumption, it was concluded that the risk of major adverse CV events may depend not only on the dosage of alcohol consumed but also on the differences in the time alcohol is consumed, being higher the risk at one hour consumption, event in moderate amounts, and decreasing at 24 h or one week [14]. In the same meta-analysis, the authors observed a U-shaped association between the amount of alcohol consumed and the acute and chronic risk of AMI, with the highest protection with approximately 2 drinks in one day and the highest risk at 9 drinks in a day [14]. 

## 4. Sex Differences 

As depicted in Table 1, the beneficial amount of alcohol intake is approximately double in men than in women because of sex differences associated with alcohol metabolism and, thus, sex differences in health effects of alcohol consumption. In both men and women, regular moderate alcohol intake is associated with decreased risk of CVD; however, women absorb alcohol differently because of their lower body water content and generally smaller stature [37]. In addition, because of their lower gastric alcohol dehydrogenase activity [38,39] leading to lower alcohol metabolism and clearance [40], they present higher vulnerability to the organ injury effects of acute and chronic alcohol consumption such as alcoholic cardiomyopathy [41]. In fact, women are more sensitive to the adverse effects at a similar level of alcohol consumption [42]. Moreover, it is well documented that CVD differs significantly between men and women at fertile ages in which women are more protected, whereas, at menopause, CVD incidence tends to be similar between women and men [43]. Cardiovascular disease is the main cause of mortality in women ≥65 years old, and, at those ages, CVD kills about 51% women and 42% men, at least in Europe [40]. Therefore, the cardioprotective effects of moderate alcohol consumption will be more visible in older adults who are at increased CV risk. 

Some authors have observed sex-specific differences in the association between alcohol intake, heart failure, and non-CV mortality. Women who consumed alcohol in a heavy and moderate manner showed decreased risk of heart failure compared to abstainers [29]. In parallel, other researchers have found that, whereas in men, the frequency of alcohol intake, independently of the amount consumed, was inversely associated with the risk of coronary heart disease, in women, the amount of alcohol but not the frequency was responsible for such an association [44]. In addition, the authors also observed that moderate alcohol consumption reduced the risk of hypertension in women but increased the risk of hypertension in men [44]. In a meta-analysis of the results reported by 14 intervention studies, alcohol consumption was associated with reduced fasting insulin concentrations and improved insulin sensitivity among women only [45]. In this line, a meta-analysis comprising over 2 million individuals supported the assumption that alcohol consumption in women decreased the risk of type 2 diabetes, independently of the amount consumed, compared to life-long and current abstainers, whereas in men, such a protective effect was not observed [46]. Sex-specific differences in the effects of alcohol in atrial fibrillation have been described as well. In men, even moderate alcohol consumption is associated with increased risk of atrial fibrillation, whereas moderate alcohol consumption is not associated to increased risk of atrial fibrillation in women [47,48,49]. However, a prospective study of Swedish men and women did not observe sex differences in this regard [50]. Oppositely, alcohol consumption was positively associated with increased blood pressure only in men in a French cohort [51]. The inverse relationship between moderate alcohol consumption and overall mortality has been shown to be apparent with up to 3 drinks per day in men but only up to 2 drinks per day in women, while the maximum risk reduction was similar in men and women [52], suggesting that women are more exposed than men to death for any cause at moderate to heavy levels of alcohol consumption, probably because of the increased risk of cancer. In fact, it has been shown that women who consume alcohol in moderate amounts have a 10% increased relative risk of overall mortality compared with men [53].

Besides intrinsic sex differences in the metabolism and biological effects of alcohol, patterns of alcohol consumption are different between men and women. Women tend to drink less amounts of alcohol than men [7,54], which, in turn, may be related to the differences in sensitivity to alcohol. Moreover, life-long abstainers and teetotalers are women to a higher extent [7,55,56,57,58], probably because of religious and cultural character traits and social inequalities. In addition, as premenopausal women have a low incidence of CVD, the benefits of alcohol on total mortality may be blunted. This should also be considered when interpreting the relationship between alcohol and health from a global perspective, and also considering that both in epidemiological and clinical trials women have been underrepresented. 

## 5. Ethnic Differences

Unfortunately, the vast majority of epidemiological studies and clinical trials linking alcohol consumption and CVD are performed in caucasic males and females to a lesser extent. Therefore, the results may not be extrapolable to non-White, non-caucasic populations.

Whereas different ethnicities may show different drinking patterns [59] and lifestyle features, the protective effects associated to low/moderate alcohol intake differ substantially among ethnicities and cultures, and also because of genetic differences in alcohol metabolizing enzymes [60]. The lower risk of all-cause mortality has been found only in White and Hispanic [61] but not in Black [62], Indian [3] or Chinese cohorts [63]. These differences have also been observed for the risk of coronary heart disease in which alcohol intake, independently of the amount consumed, was found to be protective for White but not for Black people [64]. 

Asian populations appear to have increased risk of hypertension and ischemic and hemorrhagic stroke [6] at the same amount of alcohol consumption compared to non-Asian populations [65,66], and, in a Japanese cohort, the cardioprotective effects of moderate alcohol consumption was more apparent in non-obese subjects [67].

Besides the ethnic nuances on the effects of moderate alcohol consumption in CVD, the harmful effects of abusive or heavy alcohol drinking are the same all over the globe [10,68]. In a population-based study of Korean adults, consumption of 5 or more drinks per week was associated with increased CV and non-CV mortality [69], and these findings have been supported by other epidemiologic observations in different areas such as Norway [70] and Australia [71]. However, more research is needed to guarantee the generalizability of these results across cultures and ethnicities.

## 6. Cardiovascular Disease

The hormetic effects of long-term and regular alcohol consumption in CVD will be briefly discussed below and are summarized in Table 2.

### 6.1. Intermediate Biomarkers of Cardiovascular Disease

Cardiovascular disease is a life-long, low-grade chronic inflammatory and oxidative disease, initiated by elevated low density lipoprotein (LDL) cholesterol levels and deposition in the intima forming atheromatous plaque. This plaque may eventually rupture, triggering thrombus formation which may occlude the blood vessel leading to a MACE [72]. In this setting, reduced concentrations of intermediate biomarkers of inflammation, oxidation, and coagulation as well as improving the lipid profile may decrease the risk of an eventual MACE. 

Moderate alcohol consumption seems to attenuate inflammation by modulating soluble inflammatory markers such as adiponectin [73], soluble intercellular adhesion molecule-1 [74], interleukin 10 [15] or the neutrophil-to-lymphocyte ratio [75], among others [76]. In turn, whereas alcohol is a well-known pro-oxidant agent [77], the modulation of inflammatory and oxidative biomarkers largely depends on the type of beverage consumed [76,78,79], and part of these effects may be mediated by non-alcoholic components of alcoholic beverages as is further discussed. Again, heavy drinking increases the concentration of inflammatory and oxidative parameters [80,81,82] leading to CV morbidity.

Moderate alcohol consumption decreases fibrinogen and fibrin D-dimer [74] and increases tissue plasminogen activator and plasminogen concentrations [73,83,84,85], although no effect has been observed in plasminogen activator inhibitor-1, factor VII, and von Willebrand factor concentrations [74,84]. In healthy young men and, to a lesser extent, women, alcohol consumption has been shown to inhibit platelet reactivity [86,87] without altering platelet count [88]. However, the relationship between heavy alcohol consumption and thrombosis remains blurred because of the controversial results found in different trials [89]. 

### 6.2. Classical Cardiovascular Risk Factors

It is widely accepted that alcohol consumption increases high density lipoprotein (HDL) cholesterol levels in a dose-dependent manner [73,83] in both men and women and, more specifically, the HDL2 particle and apoliprotein A-I [90]. However, the effects on LDL and triglycerides have not been completely elucidated [91]. Whereas heavy alcohol consumption is associated with hypercholesterolemia [13], the cardioprotective effect of high plasma concentrations of HDL cholesterol is currently under debate [92,93].

Low/moderate alcohol consumption has been associated with decreased incidence of type 2 diabetes compared to abstainers and heavy drinkers [94], although some studies have observed a linear inverse trend in both men and women in which even heavier drinkers had a lower risk of developing type 2 diabetes than teetotalers [95]. Other authors differ, and evidence this inverse relationship exclusively in non-Asiatic women [46]. Notwithstanding, in patients with type 2 diabetes, a reduction by two or more drinks (including total abstention) per week decreased CVD risk at 10 years by approximately 44% compared with patients who maintained their (moderate) alcohol intake [96].

Heavy alcohol consumption is one of the main reversible causes of hypertension [63]. Excessive alcohol intake is associated with a higher incidence of hypertension [58,83] and isolated diastolic hypertension [97]. In fact, it has been observed that the risk for hypertension increases linearly with alcohol consumption [65,98] with almost no minimum safe dose and even irrespective of the amount consumed [99] in both men and women, although less than 1–2 daily drinks accompanying meals may not be detrimental for blood pressure [63]. However, the alcohol–risk relationship tends to be J-shaped in women and linear in men, although certain ethnic or socioeconomic groups may be more vulnerable to hypertension induced by alcohol consumption [91].

### 6.3. Major Adverse Cardiovascular Events

In the Prospective Urban and Rural Epidemiological (PURE) study [100], which included countries across a broad range of economic levels, social circumstances and health policies, low alcohol consumers, but not moderate or former alcohol drinkers, showed an approximately 17% decrease in the incidence of CVD [101] (estimated as a composite of cardiovascular death, AMI, stroke, and heart failure) compared to teetotalers, whereas heavy alcohol drinkers showed a subtle increased risk of CVD but a high increase in the risk of death for any cause [100]. 

In probably the largest cohort study performed in this field, it was shown that abstainers had increased risk of unstable angina, AMI, ischemic stroke, heart failure, peripheral arterial disease, abdominal aortic aneurysm, and unheralded coronary death compared to moderate alcohol drinkers [29]. Moreover, low/moderate alcohol consumption is associated with decreased risk of heart failure [102], whereas heavy and former drinkers showed increased risk of fatal AMI, heart failure, cardiac arrest/sudden coronary death, and transient ischemic attack and ischemic stroke, intracerebral hemorrhage and peripheral arterial disease, and decreased risk of primary AMI and stable angina [29]. In fact, recent analyses have shown that alcohol abuse is associated with a greater risk of emergency department visits, hospitalizations and rehospitalizations for heart failure [103], and all-cause hospitalizations [104]. Whereas heavy alcohol intake is associated with an increased risk of atrial fibrillation [49] but not heart failure [105], some studies have observed an association between low–moderate alcohol intake and the risk of atrial fibrillation [47,48,106], suggesting that not only binge drinkers but also regular drinkers of moderate amounts of alcohol have an increased risk of developing atrial fibrillation. However, another study observed that moderate alcohol consumption was associated with a lower risk of heart failure [102,105] with a less pronounced association in women than in men [107] and not associated with atrial fibrillation.

Moderate alcohol intake is associated with lower levels of high-sensitivity cardiac troponin T (hs-cTnT) and N-terminal pro B-type natriuretic peptide (NT-proBNP), whereas abusive intake is associated with increased levels of these biomarkers of cardiac damage [108]. It is noteworthy that alcohol consumption is also responsible for alcoholic cardiomyopathy (as its name defines it), characterized by a dilation and impairment of the left ventricle which is a considerable risk factor for AMI and sudden death [109]. Although the number of deaths attributed to alcoholic cardiomyopathy may be overestimated, at least for adults aged 65 years or older [110], it has been shown that the estimated total lifetime dose of ethanol correlates inversely with muscular strength and ejection fraction and directly correlates with the left ventricular mass [111]. 

Alcohol consumption is inversely associated with the risk of AMI [13], even at high doses, although this observation has been shown in high- and middle-income countries [112]. A meta-analysis from 599,912 current drinkers showed an inverse and approximately log-linear association of alcohol consumption with AMI [113], although these inverse associations were possibly more pronounced for non-fatal than for fatal outcomes. Controversially, a large case-control study including 5000 participants in which lifetime alcohol drinking patterns were assessed, teetotalers and occasional (heavy) drinkers were at increased risk of AMI compared to regular drinkers [25,114]. Supporting these findings, moderate alcohol consumption has been shown protective for AMI presentation in comparison with consuming more than 3–4 daily drinks, especially in women [36]. In a large cohort or more than 11 million subjects, which used electronic health record data and were monitored for 6 years, non-drinkers had increased risk of coronary artery disease, whereas no differences were observed in moderate compared to heavy drinkers [29]. Oppositely, moderate drinkers with episodes of heavy or binge drinking presented the same risk of AMI as teetotalers [115]. 

In a specific region of Poland with the highest prevalence of CVD within the country (26% higher than the average), low–moderate alcohol consumption was related to a smaller risk of coronary disease and stroke [55]. A relatively recent meta-analysis reporting data from 1,425,513 subjects showed that, compared to non-drinkers, low alcohol consumption was associated with a 15% reduced risk of stroke. Moderate alcohol consumption was not associated with the risk of stroke, whereas heavy alcohol intake increased the risk of stroke by approximately 20% [116]. In the same meta-analysis, the authors did not observe any association between alcohol consumption and the risk of hemorrhagic stroke. However, heavy alcohol drinking was associated with increased risk of hemorrhagic stroke compared to abstainers and moderate drinkers [117]. Oppositely, heavy alcohol consumption was associated with a 26%–38% increased risk of hemorrhagic stroke for both men and women [14]. In another study, low intake of alcohol was associated with decreased risk of ischemic stroke or stroke mortality. However, no such associations have been found in moderate or heavy drinkers [116]. Moreover, low–moderate alcohol consumption has been associated with a lower risk of ischemic stroke, whereas high and heavy drinking has been associated with an increased risk [118], although in this meta-analysis, low–moderate and heavy alcohol drinking was not associated with any hemorrhagic stroke subtype, and heavy drinking was associated with increased risk of intracerebral and subarachnoid hemorrhage [118]. Conversely, a meta-analysis from 599,912 current drinkers showed a linear association between alcohol consumption and risk of fatal and non-fatal stroke [113]. Overall, the relationship between alcohol consumption and both hemorrhagic and ischemic stroke remains controversial.

In patients with coronary artery disease, moderate but not heavy alcohol consumption was associated with lower severity of the disease, quantified with the Friesinger score, compared to abstainers [56]. Along this line, heavy alcohol consumption was associated with increased prevalence of coronary artery calcium but not thoracic aortic calcium [119]. In addition, low–moderate alcohol consumption has been associated with a lower incidence of CV and all-cause mortality in patients with established CVD [63,120].

## 7. Cardiovascular and All-Cause Mortality

A meta-analysis including 1 million people observed that low–moderate alcohol consumption was inversely associated with total mortality in both men and women, while higher doses of alcohol were associated with increased mortality in a J-shaped relationship between alcohol consumption and total mortality [52]. Moreover, recently pooled data from 83 studies showed a positive and curvilinear association of alcohol consumption with all-cause mortality with the lowest risk for those consuming below 100 g per week, and these associations were similar for men and women but weaker at older ages. In addition, there was a J-shaped association for the aggregate of cardiovascular disease outcomes, and these associations were stronger for fatal than non-fatal outcomes. However, this was attributed principally to AMI. In fact, authors described a linear association between alcohol consumption and risk of coronary disease excluding AMI, fatal hypertensive disease, heart failure, and fatal aortic aneurysm. When including both never-drinkers and ex-drinkers, a U-shaped association of alcohol consumption with total CVD and all-cause mortality was reported, and the threshold for lowest risk for all-cause mortality and AMI was about 100 g per week [113]. Controversially, data from the Health Survey for England shows that the protective associations between low alcohol intake and death for any cause may be partially attributable to the reference group that was not adjusted by age and sex which are determinant factors of these associations. In fact, it appears that the associations between low alcohol intake and decreased risk of all-cause mortality are significant only in men 50–64 years and in women ≥65 years compared with never drinkers with a neutral association in other age and sex groups [121]. Supporting these findings, in a meta-analysis, occasional drinkers presented a similar relative risk for all-cause mortality than low alcohol drinkers in several models of adjustment, implying that alcohol consumption, at any amount consumed, would no longer be protective compared to occasional drinkers instead of lifetime teetotalers [112,122]. 

Low-moderate alcohol consumers showed a 20% and 25% reduced risk for all-cause and CVD mortality, respectively, compared with lifetime abstainers in subjects from the US National Health Interview Surveys, whereas heavy/binge alcohol consumption was associated with increased risk of mortality for all causes and cancer [123]. In this study, only low but not moderate alcohol consumption was associated with a reduced risk of cancer mortality [123]. In the Health Professionals Follow-up Study (HPFS), among AMI survivors, moderate alcohol consumption was associated with lower risk of all-cause and CV mortality, and the relationship between alcohol consumption and with CV and overall mortality responded to a U-shaped association [124]. In a large cohort of more than 11 million subjects, a J-shaped association for fatal and non-fatal CVD and all-cause mortality was observed, with moderate alcohol drinkers in the bottom of the J [29].

Overall, low–moderate usual alcohol intake is associated with lower risk for type 2 diabetes, stroke, heart failure, and all-cause mortality [125,126]. Despite this analysis, it is worth mentioning that excessive alcohol intake is responsible for 4% of total deaths [7,63], including cirrhosis and cancer. The risk of all-cause mortality [114] and of cancers specifically rises with increasing levels of alcohol consumption, and the level of consumption that minimizes health loss has been considered to be zero [9]. In addition, the fact that alcohol consumption has been considered “healthy for the heart” by the scientific community may have produced a justification for alcohol consumption, and consumers who understand alcohol intake as healthy for CVD have been shown to consume 1.5 times more alcohol than those who did not consider alcohol as healthy for CVD [127]. However, while the causal harmful effects of heavy alcohol intake are evident and undeniable [128], moderate alcohol consumption associates with decreased risk of AMI and CV death and even a 13% lower risk of death for any cause [101]. Although Mendelian randomization studies may be challenging these associations [66], some pitfalls must be overcome before achieving definitive conclusions [129]. Therefore, while waiting these conclusions, the benefit-risk balance of alcohol consumption should be considered individually. 

## 8. Type of Alcoholic Beverage Consumed

In general terms, fermented alcoholic beverages are the product of the fermentation of hydrolyzed sugar from cereals (beer) or fruits (wine) to alcohol by the *Saccharomyces cerevisiae* yeast. Liquors and spirits (distilled alcoholic beverages) are the product of the distillation of beer or wine. Consequently, the molecular composition of fermented (i.e., beer and wine) and distilled beverages (i.e., liquors and spirits) is very different. Fermented beverages contain a significant and declining concentration of bioactive compounds in this order: red wine > white wine > beer, namely, polyphenols [15,130], known to exert antioxidant and anti-inflammatory effects [131], of which the consumption of is associated to decreased incidence of chronic low-grade inflammatory diseases such as CVD [132] or cancer [133]. In addition, fermented beverages contain about 14%, 11%, and 5% of alcohol, for red wine, white wine, and beer, respectively. On the other hand, spirits contain approximately 35% alcohol, whereas liquors contain the same amount of alcohol with different percentages of sugar, both distilled beverages with negligible amounts of bioactive compounds such as polyphenols. Considering the differences in the composition of beer, wine, and liquors/spirits, it is plausible that their consumption elicits differential health effects, liquor and spirits being the worst in terms of bioactive components. 

Dissecting the concrete effects of each type of alcoholic beverage has been hampered in epidemiological trials because of the lack of data, the associations with socioeconomic status, beverage quality, and drinking patterns, among other potential confounding factors. Indeed, while some authors have postulated that the differential effects of fermented and distilled alcoholic beverages are the product of lifestyle differences and the pattern of alcohol consumption, several epidemiologic and clinical trials point to another direction. 

In a pooled cohort study, moderate wine drinkers had lower relative risk of overall mortality compared to non-drinkers, and moderate wine drinkers also showed lower overall mortality compared to non-wine drinkers [134]. In a three-country cohort, moderate wine consumers showed lower concentrations of intermediate markers of inflammation than beer drinkers [135]. However, subgroup analysis with spirit drinkers was not possible in this study. In women, the risk of stroke was lower in low–moderate wine drinkers compared to never drinkers and compared to low–moderate beer or spirits drinkers [136]. In another cohort, the risk of AMI appears weak in red wine drinkers, intermediate for white wine drinkers, and high for beer and spirits [13]. In this line, a recent meta-analysis has reported that associations of baseline alcohol consumption with all-cause mortality were stronger in drinkers of beer or spirits than of wine, not without warning about the potential for confounding effects [113], as beer and spirits were the predominant types of drinks consumed and, thus, most likely to be heavily/binge consumed, and also because other studies showed no relevant differences according to the type of beverage consumed [50]. In type 2 diabetic patients, moderate wine but not beer or spirit drinkers presented a 22%–23% lower risk of MACE and overall mortality compared to abstainers, with no differences in microvascular complications. However, compared to beer or spirits drinkers, wine drinkers showed no differences in the risk of MACE, although a reduced mortality trend was observed [101]. 

Besides alcohol, wine, specifically red wine, contains high amounts of polyphenols which have been shown to have metabolic and cardioprotective effects in a non-additive fashion to alcohol by lowering plasma concentrations of pro-oxidant and inflammatory molecules, leukocyte adhesion molecules, and improving homeostasis model assessment of insulin resistance values (HOMA-IR) and blood pressure [15,16,79,137,138]. Although in lower amounts, beer also contains polyphenols and other bioactive compounds [130]. In healthy overweight individuals, moderate alcoholic and non-alcoholic beer consumption increased the antioxidant capacity of HDL [139], and in high CV risk men, the non-alcoholic fraction of beer reduced proinflammatory cellular and soluble biomarkers involved in atherosclerosis progression [140]. A relatively recent international consensus document on the health effects of beer concluded that moderate consumption of fermented beverages (i.e., wine and beer) confer greater cardiovascular protection than spirits because of their non-alcoholic components (mainly polyphenols) [141]. This should be emphasized in the context of a safe and moderate consumption considering the fact that worldwide approximately 45% of alcohol is consumed in the form of spirits, 34% in the form of beer, and only about 12% in the form of wine [7]. However, despite the fact that wine and beer improve the profile of intermediate CV biomarkers, the question whether the risk of MACE is lower for wine and/or beer consumers compared to spirits consumers is still open and difficult to assess because drinkers often do not consume a single type of beverage.

## 9. Conclusions

The relationship between alcohol consumption and CVD appears in general terms biphasic, being protective at low and moderate amounts and detrimental at high intakes, even when occasionally consumed. Although several authors defend that the harmful effects of alcohol, even at low amounts, outweigh their benefits [142,143], current evidence supports that low amounts of alcohol are safe and beneficial for the CV system.

As per evident ethical and logistical reasons, the main body of evidence relies on epidemiological studies of associative nature which carry several limitations such as the quantification of alcohol consumption. In the future, these studies should include reliable measurements of biological biomarkers of alcohol exposure such as urinary ethyl glucuronide [144] which may better reflect short-term and habitual alcohol consumption than self-reported intake and should also include repeated measures over time.

It is worth mentioning that, despite the cardioprotective effects derived from low/moderate alcohol consumption, these benefits may be weighed against the potential harms from an individual perspective and addressing serious issues such as the propensity to alcohol dependence and collateral social harms, genetic vulnerability, pregnancy or even the family history of cancer. On the other hand, heavy and binge alcohol consumption should be categorically discouraged without any exception or pretext. Along this line, national and international guidelines should be better implemented and updated. 

Notwithstanding, alcohol consumption is increasing worldwide and consumed by about half of the population over 15 years of age [7]. As no large randomized trials have been able to be performed at the moment, from a public health perspective, several questions remain open: (1) Which daily amount of alcohol consumption can be considered as safe and truly cardioprotective? (2) Which type of alcoholic beverage is really more beneficial? (3) Do the effects of alcohol vary according to the region and socioeconomic status of the countries and because of genetic and ethnical traits? (4) Are the effects of alcohol consumption specific at different ages? (5) Do sex-specific differences in the pathophysiological effects of alcohol consumption disappear at a certain age? To summarize: to drink or not to drink? This question was launched in 2007 [145] and remains unanswered 13 years after. This is of especial importance considering the fact that the majority of disease endpoints attributable to alcohol consumption are also associated with aging [146], and also considering that the main body of evidence relies in countries with the highest life expectancy. Meanwhile, as alcohol consumption is part of the lifestyle of several cultures, it would be wise to suggest low–moderate alcohol consumption among current drinkers and never recommending drinking in order to improve health outcomes. 

## Figures and Tables

**Table 1:** Definition of drinking levels according to the US National Institute on Alcohol Abuse and Alcoholism.

Drinking Level	Number of Drinks ^1^	Amount of Alcohol
Low-risk consumption	≤3 drinks on a single day for women	≤32 g/single day (occasional)
	≤7 drinks/week for women	≤14 g/day (regular)
	≤4 drinks on a single day for men	≤46 g/single day (occasional)
	≤14 drinks/week for men	≤28 g/day (regular)
Moderate	≤1 drink/day for women	≤14 g/day (regular)
	≤2 drink/day for men	≤28 g/day (regular)
Binge	≥4 drinks in 2 h for women	56 g in one occasion
	≥5 drinks in 2 h for men	70 g in one occasion
Heavy	≥5 binge drinking days in a month	≥280 g in a month

^1^ A drink is defined as 14 g of pure alcohol.

**Table 2:** Effects of alcohol consumption in cardiovascular risk biomarkers, classical risk factors, and major events according to the amount of alcohol consumed.

Cardiovascular Parameter	Low/Moderate Alcohol Consumption	Heavy/Binge Drinking
Intermediate biomarkers		
Inflammation	+ ^1^	−
Oxidation	± ^1^	−
Thrombosis	+	±
Classical risk factors		
Lipid profile	+	−
Glucose metabolism	+	±
Blood pressure	−	−
Major adverse cardiovascular events		
Acute myocardial infarction	+	±
Stroke	+	−
Cardiovascular mortality	+	−

^1^ Differences observed depending on the type of alcoholic beverage consumed. “+” indicates protective effects (inverse association); “−” denotes detrimental effects (positive association); and “±” signifies neutral effects (lack of association) or inconclusive/contradictory results.
